# Trail-Based Search for Efficient Event Report to Mobile Actors in Wireless Sensor and Actor Networks †

**DOI:** 10.3390/s17112468

**Published:** 2017-10-27

**Authors:** Zhezhuang Xu, Guanglun Liu, Haotian Yan, Bin Cheng, Feilong Lin

**Affiliations:** 1 School of Electrical Engineering and Automation, Fuzhou University, Fuzhou 350116, China; n150120060@fzu.edu.cn; 2 Department of Electronic and Information Engineering, Hong Kong Polytechnic University, Hong Kong 999077, China; haotian.yan@connect.polyu.hk; 3 Wireless Information Network Laboratory (WINLAB), Rutgers University, North Brunswick, NJ 08902, USA; cb3974@winlab.rutgers.edu; 4 College of Mathematics, Physics and Information Engineering, Zhejiang Normal University, Jinhua 321004, China; bruce_lin@zjnu.edu.cn

**Keywords:** trail-based search, event report transmission, mobility management, routing, wireless sensor and actor networks

## Abstract

In wireless sensor and actor networks, when an event is detected, the sensor node needs to transmit an event report to inform the actor. Since the actor moves in the network to execute missions, its location is always unavailable to the sensor nodes. A popular solution is the search strategy that can forward the data to a node without its location information. However, most existing works have not considered the mobility of the node, and thus generate significant energy consumption or transmission delay. In this paper, we propose the trail-based search (TS) strategy that takes advantage of actor’s mobility to improve the search efficiency. The main idea of TS is that, when the actor moves in the network, it can leave its *trail* composed of continuous footprints. The search packet with the event report is transmitted in the network to search the actor or its footprints. Once an *effective footprint* is discovered, the packet will be forwarded along the trail until it is received by the actor. Moreover, we derive the condition to guarantee the trail connectivity, and propose the redundancy reduction scheme based on TS (TS-R) to reduce nontrivial transmission redundancy that is generated by the trail. The theoretical and numerical analysis is provided to prove the efficiency of TS. Compared with the well-known expanding ring search (ERS), TS significantly reduces the energy consumption and search delay.

## 1. Introduction

Wireless sensor and actor networks (WSANs) [1,2,3] are composed of a large number of sensor nodes and several mobile actors. The mobile actors move in a large area to execute missions, such as rescuing survivors or fire surveillance. The sensor nodes monitor the area and provide supports for the actors. When an event of interest is detected, the sensor node should forward an event report to inform the actor. For the mobility of the actor, its location information is always unavailable for the sensor node, which makes the event report transmission a challenging issue.

Location service and search strategy are two popular solutions to solve this problem [4]. In the location service, the actor periodically updates its location information to a subset of sensor nodes and the source can query these selected nodes to obtain the location of the actor. On the other hand, the search strategy is generally developed based on flooding which can forward the packets without the location of targets [5,6,7]. Compared with the location service, the search strategy has no overhead when there is no event occurs. Therefore, the search strategy is a reasonable solution for event report transmission to mobile actors.

Expanding ring search (ERS) is a predominant search strategy [5]. In ERS, the search packets are broadcast and propagated in the network with a preset time-to-live (TTL) value. The TTL value decreases at every time the packet is relayed. When TTL value expires, the propagation stops. This process is called a *search attempt*. If the target locates within this area, it will reply an acknowledgement (ACK) packet back to the source. Otherwise, the source will eventually time out and initiate another search attempt that covers a larger network area. The setting of TTL reduces the energy consumption compared with simply flooding the search packets all over the network. However, an unsuccessful search attempt results in repetitive broadcasting which increases both search delay and energy consumption.

**Figure 1:**
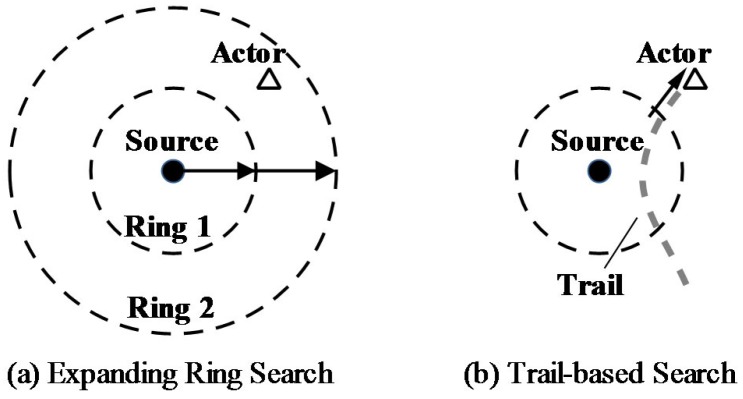
Expanding Ring Search and Trail-based Search.

In this paper, we focus on the search strategy design based on ERS. We propose a trail-based search (TS) strategy which takes advantage of actor mobility to improve search efficiency. The basic idea of TS is that: when the actor moves in the area, it leaves its *footprints* by broadcasting a tiny beacon packet. The footprints are recorded by nearby sensor nodes, and the footprints form a continuous *trail* in the sensor network. In this case, the search attempt is successful when either the actor or its footprints are discovered. When a footprint is discovered, the search packet will be forwarded along the trail until it is received by the actor. As shown in Figure 1, the trail is much easier to be found than the actor itself, thus the trail-based search may greatly reduce the search delay and energy consumption.

Specifically, this paper has the following contributions:

(1) A distributed mechanism is proposed to generate the actor’s trail. The footprint information is recorded locally in each sensor node, and it is not required to be forwarded by sensor nodes. Therefore, the overhead is extremely low. Moreover, we formally derive the condition to keep the connectivity of the trail which is important to guarantee reliable event report transmission.
(2) We propose the trail-based search (TS) strategy which uses the trail information to improve search efficiency. TS strategy is composed of two phases: the search phase and the chase phase. The search phase is similar to ERS strategy, except that the search attempt is successful when either the actor or its footprints are discovered. In the chase phase, the packets are forwarded along the trail to chase the actor. Moreover, the *effective footprint* is proposed to balance the efficiency between the search phase and the chase phase.
(3) The transmission redundancy generated by the trail is evaluated in this paper. Since the footprints are generated by the broadcast from the actor, multiple sensor nodes can receive the same footprints simultaneously. Thus in TS, there may be multiple nodes initiate the chase phase simultaneously and generate transmission redundancy. The numerical analysis proves that the transmission redundancy is non-trivial in TS. Then we propose a redundancy reduction scheme for TS (TS-R) which uses local trail information to reduce the transmission redundancy in the chase phase.
(4) Theoretical and experimental analysis is provided to study the performance of TS. The results prove that, TS significantly reduces the energy consumption and search delay by comparing with ERS and XYLS. Moreover, TS scales well to the scenarios with multiple actors due to the flooding-based process in the search phase.

The remainder of this paper is organized as follows. Section 2 provides a brief survey of related works. The details of TS are given in Section 3. Then we study the transmission redundancy in TS and introduce TS-R scheme in Section 4. The theoretical analysis is provided in Section 5. In Section 6, simulations are carried out to evaluate the performance of TS and TS-R. Finally, Section 7 concludes this paper.

## 2. Related Work

In this section, we provides a brief introduction about related works that handle node mobility in wireless sensor and actor networks. Generally, these works can be divided into two categories: location service and search strategy.

### 2.1. Location Service

Location service is a popular solution to handle mobility in ad-hoc and sensor networks. In location service, the actor updates its location information to a subset of sensor nodes and the source can query these selected nodes to obtain the location of the actor. These selected nodes are defined as location servers and connect the source and the actor. The location service can be further divided into *quorum-based* and *home-based* protocols based on the methods to construct the rendezvous area.

In the quorum-based approach, such as XYLS [8], the actor updates its location to a vertical area, and the source sends its location query to a horizontal area. The intersection of these two areas becomes the rendezvous area where the query will be satisfied. The Local Update-based Routing Protocol (LURP) proposed by Wang et al. [9] provides an algorithm which constrains the location updating within a local area. When the actor moves out of the local area, another area is built. The Adaptive Location Update-based Routing Protocol (ALURP) [10] makes an improvement compared with the LURP protocol. In ALURP, the mobile sink adaptively adjusts its location updating range when it moves in the destination area. MLS in [11] takes advantage of the fact that in most applications, the actor knows its own mobility strategy, such as its destination, trajectory and speed. Therefore, the actor can share its mobility strategy in the update packet to improve the performance of location service. The authors in [12] adopt quorum-based schemes to real irregular fields with voids as well as non-rectangular shapes and propose an energy-balanced Sink Location Service scheme.

In home-based protocols, such as GLS [13] and GHLS [14], the rendezvous area is selected via a public hash function. The input of the hash function is a node ID, and the output can be either node IDs or geographic locations. GLS (Grid Location Service) and HLS (Hierarchical Location Service) coupled to the GPSR routing protocol (Greedy Perimeter Stateless Routing) are compared in [15]. This paper presents the efficiency and the scalability performance study of both GLS and HLS and analyze their applicable scenes respectively. The Line-based data dissemination protocol (LBDD) [16] uses a virtual vertical line or strip area constructed in the central field of the network which can divide the whole network into two equal parts. The source transmits the data packet towards the virtual area. When the actor requests event reports, it also sends a query to this rendezvous area. Then it travels linearly until it encounters the node storing the packets. Yuanyuan Zeng et al. [17] proposed a directional routing and scheduling scheme (DRSS). In DRSS the source could hold or relay data to a predetermined area called rendezvous area. These event packets are stored in this area until a mobile sink is nearby to collect.

The major disadvantage of location service is the requirement of periodical location update which does not consider the occurrence of the events. The overhead of location update may waste considerable energy when there is no event occurs. Moreover, the packets should be forwarded to the rendezvous area first and then to the mobile actor, which increases the transmission delay and generates unbalanced energy consumption. At last, the location of actors may be hard to be obtained in many applications [18].

### 2.2. Search Strategy

Search strategy is a reasonable solution for event report transmission since it has no overhead when there is no event appears. It can forward the packets to the target without its location [5,6,7,19]. Expanding ring search (ERS) [5] is a predominant search strategy based on flooding. In ERS, the search packets are broadcast and propagated in the network with a preset time-to-live (TTL) value. The TTL value deceases at every time the packet is relayed. When TTL value expires, the propagation stops. This process is called a *search attempt*. If the target locates within this area, it will reply an acknowledgement (ACK) packet back to the source. Otherwise, the source will eventually time out and initiate another search attempt that covers a larger network area. The setting of TTL reduces the energy consumption compared with simply flooding the search packets all over the network.

There are some other flooding-based search strategies. The authors in [20] consider the recontamination problem that may lead to packets loss in the duty-cycled WSAN, and then propose the ballooning algorithm which builds a blocking and expanding circle during the flooding process to prevent recontamination and hence ensures the deliver rate. Concentrating on the data congestion and overloading that affect the overall performance of network, the research in [21] proposes the methodology that discovers the nearest neighbor and route to reduce the flooding and energy consumption in the network. Striped-flooding [7] adopts the striped deployment pattern in the design of flooding process, and thus improves the energy efficiency and scalability with minor impact on the deliver rate and search delay.

The major disadvantage of flooding-based search strategy is the considerable energy consumption in the flooding process. Therefore, there are some research works design search paths to reduce the energy consumption. IRS [6] routes the search packet along a set of trajectories called rays that maximizes the likelihood of discovering the target information by consuming least amount of energy. The rays are organized such that if the search packet travels along all these rays, then the entire terrain area will be covered by its transmissions while minimizing the overlap of these transmissions. The paper in [22] proposes a forwarding protocol based on biased random walks where nodes only use local information about neighbors and their next active period to make forwarding decisions. Although these works reduce the energy consumption in the search process, they generally increase the search delay which can not be tolerated in the event report transmission.

### 2.3. Trail-Like Routing

Some approaches have been proposed to take advantage of actor mobility to improve routing efficiency in ad hoc networks. In [23,24], the transmission path along the trail has been proved to be asymptotically optimal, and scales as the shortest path. HLLS in [25] proposes a history information based light location service, using the temporal relationship among historical locations of mobile nodes in the network. In HLLS, location information of mobile nodes is propagated by Hello beacons locally, and the location query is performed with the aid of historical locations. In this way, HLLS can eliminate the tremendous periodical location updates and significantly reduce the overhead for location service. The eTrail [26] proposes a clustered trail-based protocol. When the actor broadcasts its location information, it does not just broadcast to its neighbor nodes, but a cluster with a larger area. The transmission path in a cluster is also determined when the actor makes this location update. However, all these work do not consider the characteristics of event report transmission in WSAN and the challenges in searching the trail, which are mainly addressed in our research.

Compared with related works, the TS protocol proposed in this paper distinguishes them in the following aspects: (1) TS is based on the search algorithm, which performs better than location service in the event report transmission. (2) TS takes advantage of the trail information to improve search efficiency which has not been considered in existing search algorithms. (3) This paper makes in-depth study about the trail including its connectivity and redundancy.

## 3. Trail-Based Search Strategy

### 3.1. Problem Statement

In this paper, we consider the wireless sensor and actor network which has the following properties.

- The network area is a square with side length *H*. There are $N_{S}$ static sensor nodes deployed in the network, and one mobile actor moves in the network to perform actions. The mobility of the actor follows Random Waypoint model.
- The transmission range of both the sensor nodes and the actor is denoted as *R*, and the connectivity of the network is guaranteed with given *R*.
- Two sensor nodes *i* and *j* are *neighbors* if $d_{i,j}\leq R$. We define *neighbor table*, denoted as N(i), as the set of nodes in the node *i*’s transmission range. Each sensor node in the area obtains a neighbor table when the network is initialized.
- The time is divided into slots.

In the network stated above, we consider the problem of searching the mobile actor. When a sensor node detects an event, it becomes a *source* and generates a *search packet* which includes an event report. Generally, the actors’ mobility pattern is not available to the sensor nodes. Therefore, the search packet has to be forwarded to the mobile actor without its location information.

Our goal is to design a search strategy that exploits the trail information of the actor to improve the search efficiency. In the rest of this section, a distributed mechanism is firstly proposed to maintain the actor’s trail with low overhead. Then we introduce the details of trail-based search (TS) strategy which is composed of two phases: the search phase and the chase phase.

### 3.2. Trail Maintenance

To leave its trail on the sensor network, the actor should periodically broadcast its location information when it moves in the network. The period is defined as the update interval of the actor $T_{u}$. The sensor node records the time slot when it receives the location information. The time stamp is defined as *footprint*, denoted by θ. The footprint is updated only when the sensor node receives a new update from the actor. Moreover, there is no need for sensor nodes to exchange their footprint information. Thus, the overhead to maintain the trail is extremely low.

When the network has been running for some time, the actor may leave a long trail in the network. A long trail increases the possibility of finding the footprints, and thus reduces the energy cost in the search phase. However, the efficiency of the chase phase could be compromised. It is because that the trail may be too long to be an efficient path for chasing. To address this issue, we introduce a new variable *age*, denoted as α=t−θ, where *t* is the current time slot. A footprint with smaller age implies that the actor appears around more recently. In TS, the packet ignores all the footprints that have α>ε, where ε is the threshold to classify ages. The threshold ε can be used to balance the efficiency between the search phase and the chase phase.

### 3.3. Search Phase

The trail-based search strategy consists of two phases: search phase and chase phase. The basic process of the search phase is the same as ERS. The source generates a search packet with a preset time-to-live (TTL) integer, and then broadcasts the packet to its neighbor nodes. Meanwhile, the source initiates a waiting timer $T_{w}$ to wait for the acknowledgment from the actor. The nodes that receive the search packet continue to broadcast this packet with a decrease in TTL value. The forwarding stops when TTL=0. If the actor locates within the transmission range of any sensor node that broadcasts the search packet, it will receive this packet, and the search attempt is successful. Then the actor sends an acknowledgment (ACK) packet back to the source. The waiting timer $T_{w}$ should be set long enough to ensure the ACK packet can be received by the source before it terminates. If no ACK packet is received when $T_{w}$ terminates, the source will initiate another search attempt with a larger TTL value and search for a larger search area.

Different from ERS, TS searches not only the actor, but also its footprints. We define the *host* as the node that receives the search packet and obtains a footprint with $\alpha_{h}\leq \varepsilon$. If the host decides to broadcast the search packet, it will record its footprint into the packet. The footprint recorded in the packet is denoted as $\theta_{p}$. Generally, the actor may leave a long trail in the network which increases the success rate of search attempt in TS. Nevertheless, it also results in search redundancy that multiple nodes discover the footprints simultaneously in the search phase. To solve this problem, we propose the definition of the *effective footprint* as follows.

**Definition** **1** (Effective Footprint)**.** Given a host that receives the search packet with $\theta_{p}$ and TTL=0, its footprint $\theta_{h}$ is an effective footprint when it satisfies $\alpha_{h}<\varepsilon$ and $\alpha_{h}<\alpha_{p}$ simultaneously.

**Figure 2:**
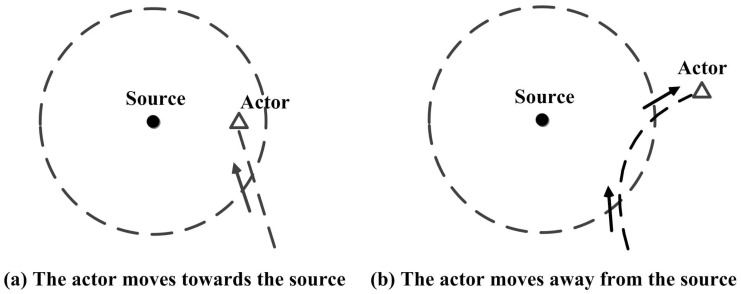
The scenarios that the packet discovers the footprints.

In the search phase, the host that obtains an effective footprint should transmit an ACK packet back to the source and initiate the chase phase. The definition of the effective footprint is derived based on the following observation. If the footprint is discovered within the search area, there will be two scenarios. The first one is that the actor is moving inside the search area as shown in Figure 2a. In this case, the actor will be discovered in the search phase, thus there is no need to chase the actor along the trail. Another scenario is that the actor is moving away from the source as shown in Figure 2b, such that only the footprint can be discovered in the search phase. In this case, the host should transmit an ACK packet back to the source and trigger the chase phase. As shown in Figure 2b, the actor is ensured to leave footprints on the edge of the search area (the host that receives the search packet with TTL=0). By comparing $\alpha_{h}$ and $\alpha_{p}$, the host can estimate in which direction the actor moves. If $\alpha_{h}<\alpha_{p}$, which means that the actor is moving out of the search area, the host will initiate the chase phase. The working flow of the search phase is summarized in Algorithm 1.

**Algorithm 1** Trail-based Search Strategy: Search Phase**Require:**
$\alpha_{h}$, $\alpha_{p}$, ε, *S*, ΔS1: **if** Source node **then**2: Sets up waiting timer $T_{w}$3: Broadcast the search packet with TTL=S4: **if**
$T_{w}=0$
**then**5:  **if** Receive ACK packet **then**6:   Search success;7:  else8:   Update S=S+ΔS;9:   The source generates a new search packet, return to line 2;10:  **end if**11: **else**12:  Wait for ACK packets; return to line 4;13: **end if**14: **end if**
15: **if** Sensor node that receives search packet **then**16: **if**
TTL≠0
**then**17:  TTL=TTL−1;18:  Broadcast the search packet;19: **else**20:  **if**
$\alpha_{h}<\varepsilon$ AND $\alpha_{h}<\alpha_{p}$
**then**21:   Initiate the chase phase;22:   Send an ACK packet back to the source;23:  **end if**24: **end if**25: **end if**
26: **if** Actor node that receives search packet **then**27: Send an ACK packet back to the source;28: **end if**


### 3.4. Chase Phase

When an effective footprint is discovered, the chase phase is triggered. In the chase phase, the packet transmission is based on the following rules. When a host receives the packet, it checks the age of its footprint $\alpha_{h}$ and that of the footprint recorded in the packet $\alpha_{p}$. If $\alpha_{h}<\alpha_{p}$, the host will record its footprint in the packet and then broadcast the packet to its neighbors. Otherwise, the host will discard the packet. This scheme ensures that the packet is transmitted to the host with smaller α at each hop. Therefore, the packet will be propagated along the trail and eventually received by the actor.

## 4. Reduce Redundancy in Trail-Based Search

In this section, the transmission redundancy of TS is firstly evaluated by simulations. Then we introduce the redundancy reduction scheme to solve this problem.

### 4.1. Transmission Redundancy in Trail-Based Search

Due to the broadcasting manner of trail maintenance, there are multiple sensor nodes record the same footprint information that results in transmission redundancy. Although the *effective footprint* proposed in TS can significantly reduce the redundancy in the search phase, the transmission redundancy still exists in two aspects: the redundant nodes that initiate the chase phase, and the redundant nodes involved in the transmission path of the chase phase.

The simulations are executed to evaluate the transmission redundancy in the chase phase. There are 900 nodes uniformly deployed in 600 m × 600 m area. The actor moves in the area that follows random waypoint model [27] with no pause time at a speed of 1 m/s. The TTL and the increment are both set to be 4 and the update interval $T_{u}$ varies from 1 to 25 s.

**Figure 3:**
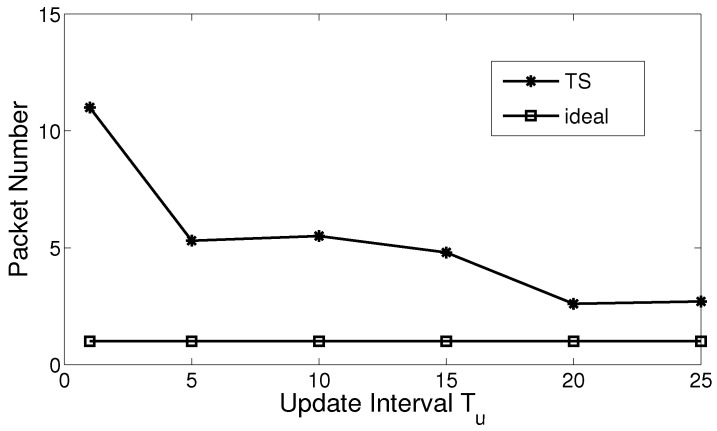
Transmission redundancy in trail-based search (TS).

We firstly obtain the average number of hops in the chase phase, and then compare it with the number of packet transmission executed by sensor nodes. Ideally, if there is no transmission redundancy, the number of packet transmission will be the same as the number of hops. However, as shown in Figure 3, the number of packet transmission are 3–5.5 times over those in the optimal scenario.

Moreover, Figure 3 shows that the transmission redundancy reduces with the growth of the update interval. The reason for this is that when the update interval is small, especially when $T_{u}\leq 5$, the actor broadcasts footprint information frequently, such that more sensor nodes will receive the footprints. It leads to considerable redundancy of the footprints, and the redundancy of the footprints will eventually result in the redundancy of packet transmission.

Although transmission redundancy can be controlled by increasing $T_{u}$, simply increasing $T_{u}$ may lead to the disconnection of the trail that impacts the deliver rate of packet transmission. Therefore, in Section 4.2, we propose a redundancy reduction scheme for TS, which has no relation with the update interval of the trail. The connectivity of the trail will be further studied in Section 5.1.

### 4.2. Redundancy Reduction Scheme

The analysis given in Section 4.1 shows that there is nontrivial transmission redundancy in the chase phase of TS. This motivates us to propose a new scheme named *TS-R* to reduce the transmission redundancy in TS.

The solution is based on a greedy strategy: selecting the node closest to the recorded location of actor as the next-hop relay at each hop in the chase phase. To realize this idea, more information is required to be saved in the trail update packet and the search packet. In the trail maintenance, the actor is required to write the coordinate of its current location into the trail update packet. Moreover, in the transmission of search packets, the host should record its location into the search packet and then broadcast it. It is important to note that, only the location of the previous hop node is recorded in the search packet, thus the overhead of TS-R is still low.

When a host finds it obtains an effective footprint, it first obtains its distance to the recorded location of the actor, denoted as $d_{h,A}$. Then it checks its neighbor table N(h). Consider each sensor node i∈N(h), the host derives node *i*’s distance to the previous hop node $d_{i,p}$ and that to the actor $d_{i,A}$. If there is no node in the neighbor table N(h) that satisfies both $d_{i,p}\leq R$ and $d_{i,A}<d_{h,A}$, the host will continue to broadcast the search packet. Otherwise, it will discard the search packet.

**Figure 4:**
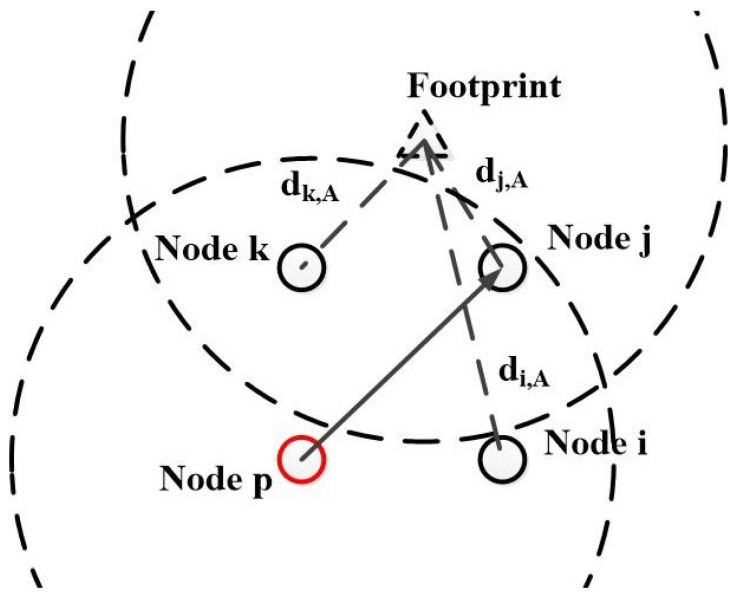
An example of data transmission in the redundancy reduction scheme based on TS (TS-R).

Figure 4 shows an example that demonstrates how TS-R reduces the transmission redundancy in the chase phase. When node *p* broadcasts the search packet, node *i*, *j*, and *k* can receive this packet. For node *i*, since $d_{i,A}>R$, it does not satisfy the condition $\alpha_{i}<\alpha_{p}$ and thus discards the packet. For node *k*, it finds that there is a node *j* in N(k) that satisfies the condition $d_{j,A}<d_{k,A}$. Thus node *k* does not forward the packet either. Only the node *j* continues to forward the search packet, because it satisfies $d_{j,p}\leq R$ and it is closer to the actor than any other nodes in both N(p) and N(j), i.e., the node *i* and *k*. Therefore, TS-R scheme can reduce the transmission redundancy in the chase phase, and improve the energy efficiency of the network. Simulation results will be provided in Section 6.2.3 to prove the efficiency of TS-R.

## 5. Theoretical Analysis

In this section, the theoretical analysis is provided to prove the efficiency of the TS strategy. Firstly, we derive the condition to keep the connectivity of the trail. Then we evaluate the performance of TS strategy in terms of search delay and energy consumption, and compare it with that of ERS.

**Figure 5:**
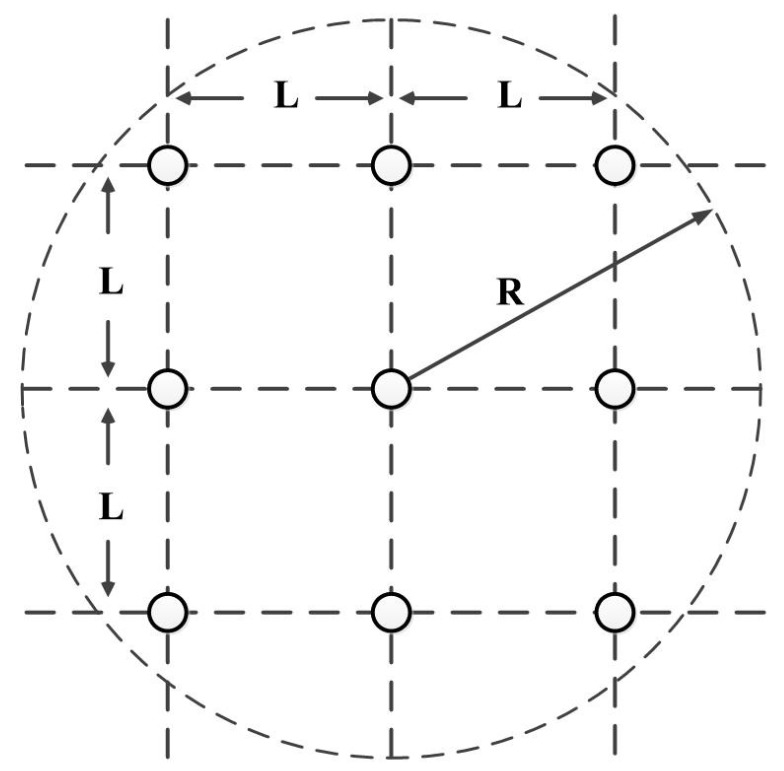
Network Model in Theoretical Analysis.

Without loss of generality, we assume that the sensor nodes are uniformly deployed that partition the network area into grids. Each grid is a L×L square, and the nodes are deployed at the corners of the grids. The transmission range of both the sensor nodes and the actor are limited to their adjacent grids (excluding diagonal grids), constrained by $R\in (\sqrt{2}L,2L)$. An example of a network is shown in Figure 5.

In the sensor network, the nodes always use duty cycle to reduce the energy consumption which results in non-trivial transmission latency. To clarify its impact on the analysis, in this section, we use the *waiting time* of packet $\tau_{P}$ and that of actor $\tau_{A}$ to formulate the transmission latency and actor mobility [20]. The $\tau_{P}$ is defined as the expected number of time slots required for one hop data transmission, and the $\tau_{A}$ is defined as the expected number of time slots required for the actor moves from one grid to any adjacent grid. The value of $\tau_{P}$ depends on the specific MAC and PHY protocol used in the sensor nodes, while the value of $\tau_{A}$ is determined by the node density and the velocity of actor’s movement.

The variables used in the analysis are summarized in Table 1.

### 5.1. Trail Connectivity

In TS, the connectivity of the trail determines the reliability of data transmission. Therefore, in this section, we derive the condition to ensure the connectivity of the trail, which can be used as the guideline of setting trail update interval.

At first, we provide the formal definition of the trail connectivity. Let $N(A_{\theta })$ denote the set of sensor nodes located in the transmission range of the actor when it updates footprint θ at time slot $t_{\theta }$.

**Definition** **2** (Trail Connectivity)**.** For any given footprint θ, the trail is connected when there exists at least one pair of sensor nodes (i,j) that satisfies $d_{i,j}\leq R$, where $i\in N(A_{\theta })$ and $j\in N(A_{\theta +1})$.

**Theorem** **1.** *With given grid length L and the speed of the actor v, the trail is ensured to be connected when the update interval $T_{u}$ satisfy,*

(1) $$T_{u}\leq \sqrt{2}\cdot \frac{L}{v}$$

**Proof.**  Let $A_{1}\left(x_{1},y_{1}\right)$ denote the coordinate of the actor at time $t_{\theta }$ and $P(x_{p},y_{p})$ denote a host on the trail that selects the next hop in its neighbor table N(P). The transmission range of sensor nodes and actors are constrained by $R\in (\sqrt{2}L,2L)$.

In TS, node *P* is closest to $\left(x_{1},y_{1}\right)$ by comparing with its neighbors, such that,

(2) $$\left\{\begin{array}{cc} x_{1}=x_{p}+a_{1} & -\frac{L}{2}\leq a_{1}\leq \frac{L}{2}\\ y_{1}=y_{p}+b_{1} & -\frac{L}{2}\leq b_{1}\leq \frac{L}{2} \end{array}\right.$$

Assume that the actor arrives at $A_{2}\left(x_{2},y_{2}\right)$ when it broadcasts the footprint information at time $t_{\theta +1}$. Without loss of generality, consider a worst case that the actor moves consistently in the diagonal direction, then we have,

(3) $$\left\{\begin{array}{cc} x_{2}=x_{1}+D_{x} & -A_{x}\leq D_{x}\leq A_{x}\\ y_{2}=y_{1}+D_{y} & -B_{y}\leq D_{y}\leq B_{y} \end{array}\right.$$

In order to guarantee the connectivity of the trail, $A_{x}$ and $B_{y}$ need to satisfy the following condition: For arbitrarily chosen $D_{x}$ and $D_{y}$, there always exists a node in N(P), denoted by $H(x_{h},y_{h})$ that meets,

(4) $$\left\{\begin{array}{cc} x_{h}=x_{2}+a_{2} & -\frac{L}{2}\leq a_{2}\leq \frac{L}{2}\\ y_{h}=y_{2}+b_{2} & -\frac{L}{2}\leq b_{2}\leq \frac{L}{2} \end{array}\right.$$

To ensure that the packet can be transmitted successfully from *P* to *H*, node *P* and *H* have to locate within the transmission range of each other. Then we can derive that,

(5) $$\left\{\begin{array}{c} x_{h}=x_{p}\pm L\\ y_{h}=y_{p}\pm L \end{array}\right.$$

It is important to note that the aforementioned conditions can guarantee the connectivity in just one hop. However, the connectivity should be guaranteed for the whole trail. According to Equation (3), we can derive the location of the actor $A_{n}$ when it broadcasts the footprint information at time $t_{\theta +n}$,

(6) $$\left\{\begin{array}{cc} x_{n}=x_{1}+n\cdot D_{x} & -A_{x}\leq D_{x}\leq A_{x}\\ y_{n}=y_{1}+n\cdot D_{y} & -B_{y}\leq D_{y}\leq B_{y} \end{array}\right.$$

The host the actor chooses at time $t_{\theta +n}$ is denoted by $K(x_{k},y_{k})$ that meets,

(7) $$\left\{\begin{array}{cc} x_{k}=x_{n}+a_{n} & -\frac{L}{2}\leq a_{n}\leq \frac{L}{2}\\ y_{k}=y_{n}+b_{n} & -\frac{L}{2}\leq b_{n}\leq \frac{L}{2} \end{array}\right.$$

(8) $$\left\{\begin{array}{c} x_{k}=x_{p}\pm n\cdot L\\ y_{k}=y_{p}\pm n\cdot L \end{array}\right.$$

Combining Equations (6)–(8),

(9) $$\left\{\begin{array}{c} -L\leq n\cdot D_{x}-n\cdot L\leq L\\ -L\leq n\cdot D_{y}-n\cdot L\leq L \end{array}\right.$$

Since the sensor nodes make updates when they receive new footprint information, the connectivity can be guaranteed when $D_{x}<L$ and $D_{y}<L$. When n→∞, the connectivity can be guaranteed by,

(10) $$\left\{\begin{array}{c} D_{x}\leq L\\ D_{y}\leq L\\ D=\sqrt{{D_{x}}^{2}+{D_{y}}^{2}}\leq \sqrt{2}\cdot L \end{array}\right.$$

Equation (1) can be directly derived by combining $T_{u}=\frac{D}{v}$ with Equation (10). The proof is completed. ☐

When the update interval $T_{u}$ satisfies Equation (1), the actor is ensured to leave a connected trail in the network. On the other hand, it is important to note that Theorem 1 provides a sufficient condition to ensure the trail connectivity. It does *not* mean that the trail is disconnected if Equation (1) is not satisfied. More analysis will be provided via simulations in Section 6.1.

### 5.2. Search Delay

This section studies the search delay of TS strategy, and compare with that of ERS. The search delay is defined as the duration from the initiation of the search phase to the moment that the actor receives the search packet. When the actor is discovered in the search phase, the performance of TS is the same as that of ERS. Thus we only evaluate their performance when the actor is discovered in the chase phase. To clarify the analysis, we assume that the second search attempt in ERS will flood the search packets all over the network.

In TS strategy, the search delay consists of two parts: the delay in the search phase $T_{s}$ and that in the chase phase $T_{c}$. The $T_{s}$ is the time for the packet that is forwarded by *S* hops, such that,

(11) $$T_{s}=S\cdot \tau_{P}$$

In the chase phase, the packet moves along the trail to chase the actor, the delay can be estimated by

(12) $$T_{c}\leq \frac{\varepsilon /\tau_{A}}{1/\tau_{P}-1/\tau_{A}}=\frac{\varepsilon \cdot \tau_{P}}{\tau_{A}-\tau_{P}}$$

where $\varepsilon /\tau_{A}$ is the maximum distance between the packet and the actor at the beginning of the chase phase, and $1/\tau_{P}-1/\tau_{A}$ indicates the velocity difference of them.

Then we have the search delay of the TS strategy,

(13) $$D_{TS}=T_{s}+T_{c}\leq S\cdot \tau_{P}+\frac{\varepsilon \cdot \tau_{P}}{\tau_{A}-\tau_{P}}$$

In ERS, when the source initiates a search attempt, it also generates a waiting timer $T_{w}$ to wait for the ACK packet. The waiting timer should satisfy $T_{w}\geq 2\cdot T_{s}$ to ensure that the second search attempt will not be initiate before the ACK packet is received. Therefore, when the first search attempts fails, the search delay of ERS can be estimated by,

(14) $$D_{ERS}=T_{w}+M\cdot \tau_{P}\geq 2\cdot S\cdot \tau_{P}+M\cdot \tau_{P}$$

where *M* is the number of hops the packet is transmitted to the actor, and M>S. In this equation, the first item represents the delay for the source to wait until it initiates another search attempt, and the second item is the delay in the second search attempt.

The difference of search delay between ERS and TS is,

(15) $$D_{ERS}-D_{TS}\geq S\cdot \tau_{P}+M\cdot \tau_{P}-\frac{\varepsilon \cdot \tau_{P}}{\tau_{A}-\tau_{P}}$$

According to Equation (15), when the following condition is satisfied, the delay of TS is shorter than that of ERS,

(16) $$M+S>\frac{\varepsilon }{\tau_{A}-\tau_{P}}$$

Generally, the packet forwarding is much faster than the actor movement, i.e., $\tau_{A}>>\tau_{p}$. With reasonable setting of ε, the search delay in TS is smaller than that in ERS.

### 5.3. Energy Consumption

We define the unit of energy consumption as the energy cost for transmitting one packet in one hop. Similar to the analysis of the search delay, we only consider the scenario that the actor is discovered in the chase phase. The energy consumption of TS strategy consists of two parts: energy cost in the search phase and that in the chase phase. In the search phase, for every host inside the ring forwards the packet one hop each, we have the energy cost in the search phase,

(17) $$E_{s}={\left(2S-1\right)}^{2}$$

In the chase phase, with the help of TS-R scheme, there is only one packet moves along the trail to chase the actor. Thus the energy consumption in the chase phase is related to the number of hops needed to discover the actor. According to Equation (12), we have the energy consumption in the chase phase,

(18) $$E_{c}=\frac{\varepsilon \cdot \tau_{P}}{\tau_{A}-\tau_{P}}\cdot \frac{1}{\tau_{P}}=\frac{\varepsilon }{\tau_{A}-\tau_{P}}$$

The energy consumption of TS strategy is

(19) $$E_{TS}=E_{s}+E_{c}={\left(2S-1\right)}^{2}+\frac{\varepsilon }{\tau_{A}-\tau_{P}}$$

In ERS strategy, the energy consumption consists of the first search attempt and the flooding all over the network. Then we have the energy consumption of ERS strategy,

(20) $$E_{ERS}={\left(2S-1\right)}^{2}+N_{s}$$

where $N_{s}$ is the number of sensor nodes in the network.

The difference between ERS and TS can be derived as,

(21) $$E_{ERS}-E_{TS}=N_{s}-\frac{\varepsilon }{\tau_{A}-\tau_{P}}$$

According to Equation (21), when the following condition is satisfied, the energy consumption of TS is smaller than that of ERS,

(22) $$N_{s}>\frac{\varepsilon }{\tau_{A}-\tau_{P}}$$

Similar to the search delay analysis in Section 5.2, this condition is easier to achieve with reasonable setting of ε. Thus the energy efficiency of TS is guaranteed in most scenarios.

## 6. Simulation Results

In this section, we evaluate the performance of TS via simulations. The performance metrics include the delivery rate, energy consumption and search delay. Moreover, the performance of TS in the WSAN with multiple actors is also studied. The performance of ERS [5] and XYLS [8] are evaluated for comparison.

The simulations are executed in the OMNeT++ network simulator [28]. In the simulations, there are 400 sensor nodes uniformly deployed in a 400 m × 400 m area. The source is randomly selected from the network. The original location of the actor is randomly selected and its mobility strategy follows the Random Waypoint model. To create an initial trail in the network, the actor movement starts 200 time slots earlier than the generation of the source. The TTL values of both TS and ERS are set to be 8 and the increment of TTL is also 8. The transmission range is 30 m. The waiting time of packet transmission $\tau_{P}$ is fixed at 1. Each simulation runs 500 times to obtain statistical results.

### 6.1. Delivery Rate

The delivery rate is defined as the ratio of the number of successfully received search packets at the mobile actor to the total number of packets generated by the source. The velocity of the actor is fixed at 3.5 m/s. The analysis in Section 5.1 has stated that the trail connectivity has tight relation with the deliver rate, and the footprint update interval $T_{u}$ needs to satisfy $T_{u}\leq \sqrt{2}\cdot \frac{L}{v}$ to guarantee the connectivity of the trail. Therefore, we study the deliver rate with different footprint update intervals.

**Figure 6:**
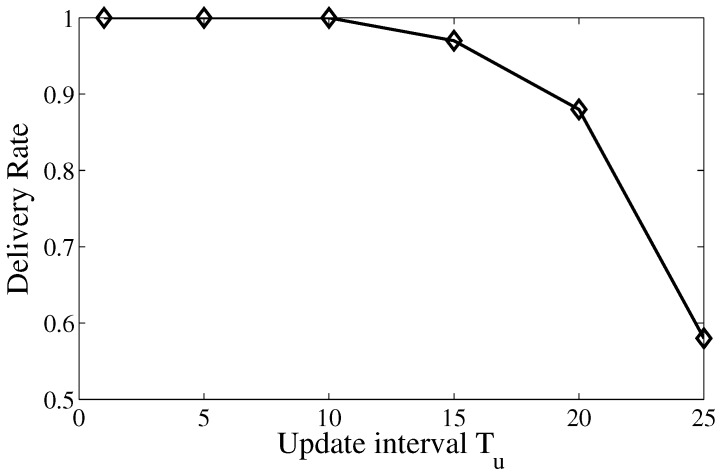
Delivery rate of TS with different footprint update interval.

As shown in Figure 6, the update interval $T_{u}$ varies from 1 to 25 s. According to Theorem 1, we obtain the upper bound of $T_{u}$ that ensures a connected trail in the given simulation setting is 8.08 s. The result shows that the delivery rate is guaranteed when $T_{u}\leq 10$. The delivery rate starts to descend when $T_{u}>10$ and drops dramatically after that. The simulation result proves the correctness of Theorem 1, and provides the guideline of setting footprint update intervals in the following simulations.

### 6.2. Energy Consumption

In TS, the energy consumption is influenced by two important factors: the number of search attempts and the transmission redundancy. The number of search attempts influences the energy consumption in the search phase and the transmission redundancy impacts the energy consumption in the chase phase. Therefore, we firstly evaluate the total energy consumption and then exploit these factors respectively. We choose two parameters: the threshold value of trail age ε and the waiting time of the actor $\tau_{A}$ to evaluate the performance and compare with ERS and XYLS.

#### 6.2.1. Total Energy Consumption

**Figure 7:**
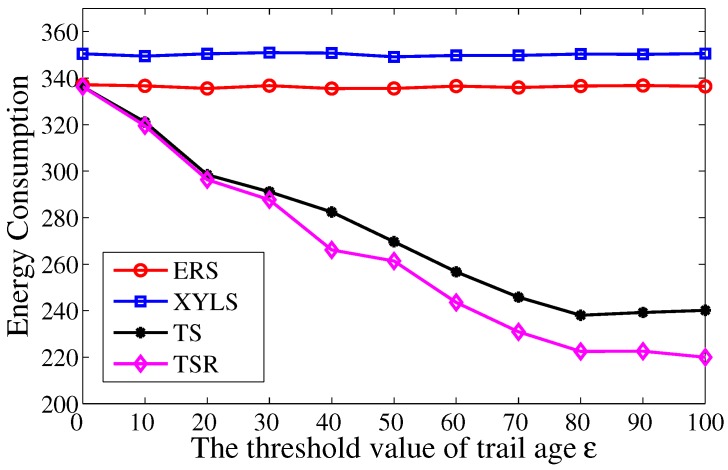
The energy consumption over the threshold value of trail age ε.

At first, the energy consumption is studied with different threshold value of trail age ε. The ε varies from 0 to 100, and the $\tau_{A}$ is fixed at 5. As shown in Figure 7, the energy consumption in TS and TS-R decreases with the growth of ε. A larger ε leads to a shorter search phase and a longer chase phase. However, the reduced energy consumption in the search phase always dominates the total energy consumption. In addition, comparing with TS, the energy consumption in TS-R drops more obviously with the growth of ε. The reason for this is that a larger ε leads to more redundancy, and the effect of redundancy reduction scheme grows in this scenario. Since there is no trail information in ERS and XYLS, their energy consumption has no relation with ε.

**Figure 8:**
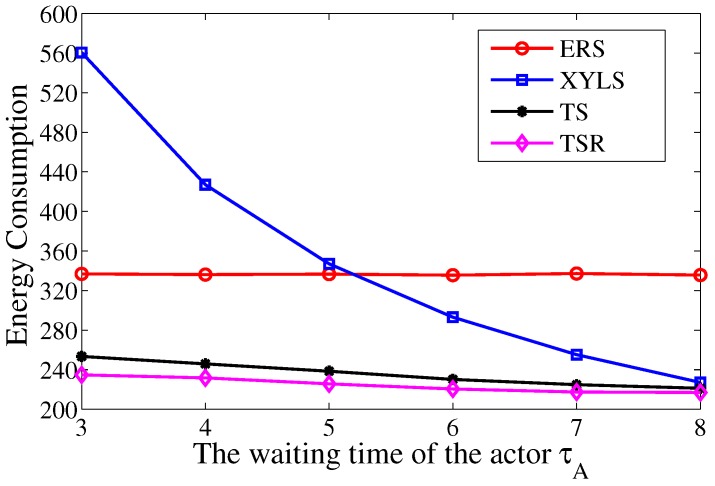
The energy consumption over the waiting time of actor $\tau_{A}$.

Then we study the energy consumption with different waiting time of actor $\tau_{A}$. The $\tau_{A}$ grows from 3 to 8, while the ε is set to be $20\times \tau_{A}$ to ensure the actor trails have the same length. Figure 8 shows that the energy consumption in TS and TS-R decreases with the growth of $\tau_{A}$. Compared with ERS, the advantage of TS increases with the growth of $\tau_{A}$. These results support the analysis given in Section 5.3. A larger $\tau_{A}$ reduces the process of the chase phase and hence reduce the energy consumption in TS. Meanwhile, the energy consumption in TS drops more than that in TS-R. The reason for this is that the transmission redundancy decreases with the growth of $\tau_{A}$. On the other hand, the energy consumption in XYLS grows dramatically when the $\tau_{A}$ decreases. The reason for this is that a smaller $\tau_{A}$ means the actor moves faster. In this case, the frequency of location update should increase to guarantee reliable transmission which leads to the growth of energy consumption. Therefore, we can conclude that the energy efficiency of TS is better than ERS and XYLS, and the advantages is larger with larger ε and smaller $\tau_{A}$.

#### 6.2.2. Number of Search Attempts

**Figure 9:**
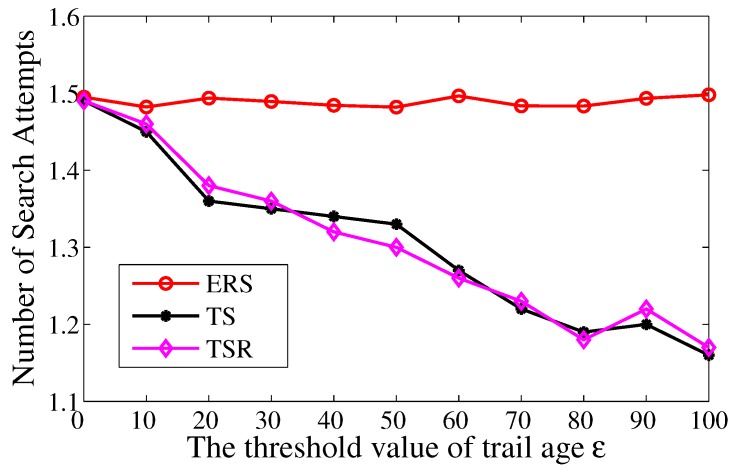
The average times of search attempts over the threshold value of trail age ε.

Figure 9 depicts the number of search attempts in the search phase. The numbers in TS and TS-R are equivalent with ERS when ε=0 and they decrease with increasing ε. This is because the feasible trail is prolonged when ε increases. Therefore, the packets have greater possibility to detect the actor’s footprints within certain search attempts, and then the number of search attempts is reduced. The numbers in TS and TS-R are almost the same because the redundancy reduction scheme has no influence on the search phase.

**Figure 10:**
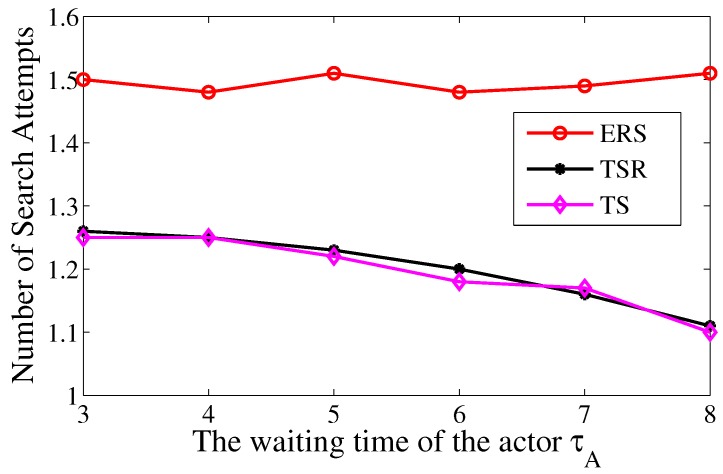
The average times of search attempts over the waiting time of actor $\tau_{A}$.

Figure 10 shows that $\tau_{A}$ has minor influence on the numbers of search attempts in TS and TS-R. The reason for this is that the value of $\tau_{A}$ mainly impacts on the chase phase and has minor influence on the search phase. Thus the number of search attempts in TS and TS-R are almost the same, and both of them has better performance compared with ERS with a certain ε.

#### 6.2.3. Transmission Redundancy

In this subsection, we concentrate the transmission redundancy and verify that TS-R can effectively reduce the redundancy. A new variable *redundancy ratio* is introduced to quantify the transmission redundancy in the chase phase, denoted as γ.

(23) $$\gamma =\frac{N}{n}$$

where *N* is the packets transmitted in the chase phase, and *n* is the number of hops on the trail in the chase phase. Ideally, if there is no transmission redundancy, the number of transmitted packets will be the same as the number of hops, i.e., γ=1. If γ is a large value, it implies that there exists non-trivial transmission redundancy. The update interval varies from 1 s to 6 s.

**Figure 11:**
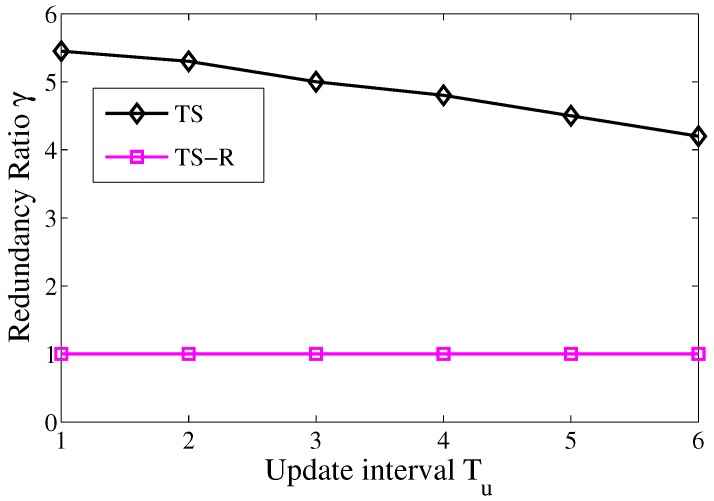
Redundancy ratio of TS-R.

As shown in Figure 11, the redundancy ratio γ of TS-R is equal to 1. On the other hand, the γ can be up to 5.46 in the TS without redundancy reduction scheme. The redundancy reduction scheme significantly reduces the number of hosts that are involved the chase phase and thus eliminates packet transmission redundancy in the network. This experiment verifies the effectiveness of TS-R.

### 6.3. Search Delay

In this section, we study the search delay of TS, and compare it with that of ERS and XYLS. The search delay is defined as the duration from the time that the source broadcast the search packet to the time that the actor receives the search packet.

**Figure 12:**
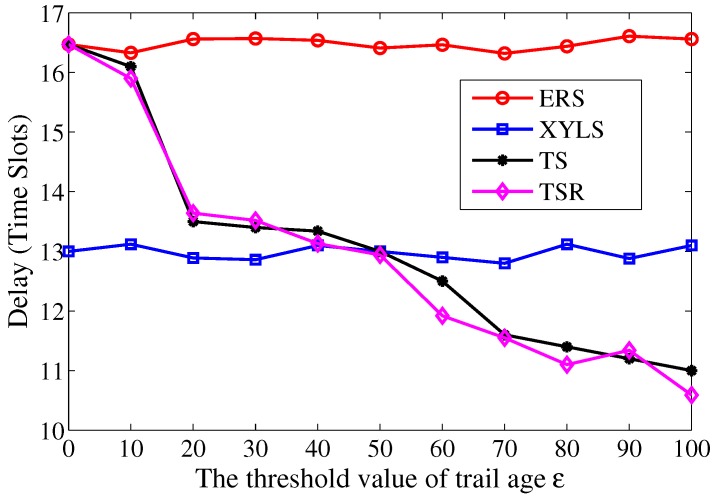
The search delay over the threshold value of trail age ε.

At first, the performance of search delay is studied with different threshold value of trail age ε. The ε varies from 0 to 100, and the $\tau_{A}$ is fixed at 5. As shown in Figure 12, with the help of the trail, the search delay in TS and TS-R are less than that in ERS. Moreover, the search delay in TS and TS-R decreases with the growth of ε. The reason for this is that, when the threshold ε increases, the number of search attempt decreases, which further results in a reduced delay. On the other hand, the search delay in XYLS is lower than that in ERS. It is because the failure of search attempt in ERS greatly increases the search delay. Nevertheless, TS has less search delay than XYLS when ε>50, since the number of search attempts in TS is smaller with a longer trail.

**Figure 13:**
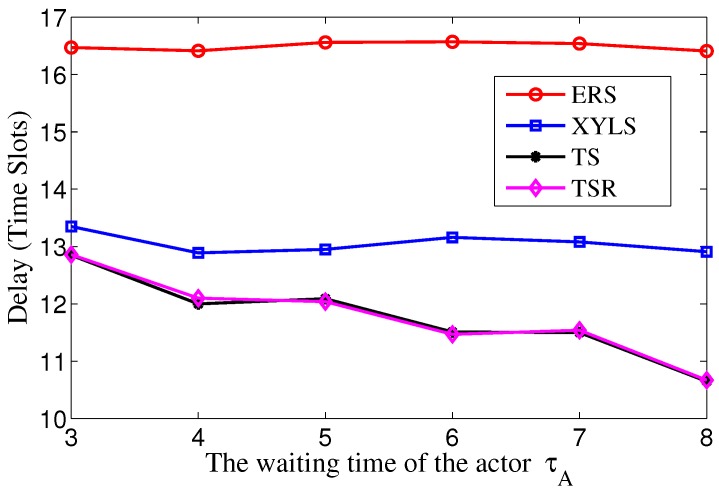
The search delay over the waiting time of actor $\tau_{A}$.

Then we study the search delay with different waiting time of actor $\tau_{A}$. The $\tau_{A}$ grows from 3 to 8, while the ε is set to be $20\times \tau_{A}$ to ensure the actor trails have the same length. As shown in Figure 13, in TS and TS-R, the search delay decreases as $\tau_{A}$ increases. We have proved in Equation (15) that the difference between the delays in ERS and TS will increase with the growth of $\tau_{A}$. The results in Figure 13 verify our analysis. A larger $\tau_{A}$ means the actor moves slower and the packet can catch up with the actor more quickly. The redundancy reduction scheme has no influence on the search delay, thus the search delay in TS and TS-R are almost the same.

### 6.4. Multiple Actors

In this section, we study the performance of TS in the scenario with multiple actors. The energy consumption and search delay are recorded when all the actors receive the search packet. The number of actors grows from 1 to 6. The $\tau_{A}$ is fixed at 5, and the ε is set as 100.

**Figure 14:**
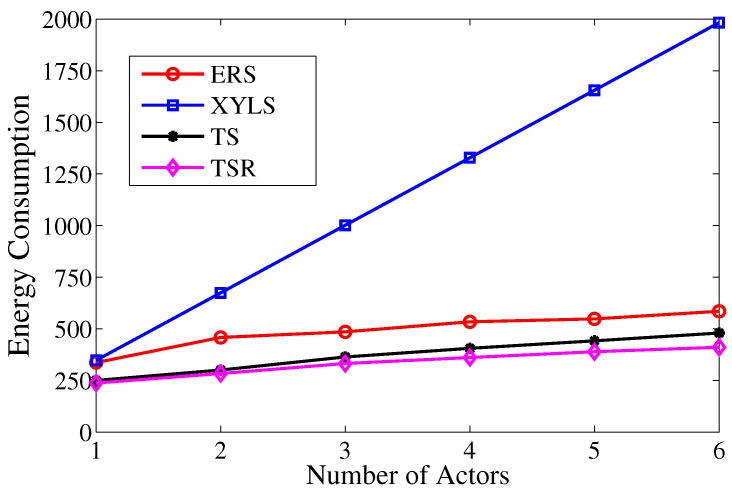
The energy consumption over the number of actors.

As depicted in Figure 14, the energy consumption in XYLS rises rapidly with the increase of actors. The reason for this is that every actor has to execute location update individually in XYLS. Moreover, the overhead of location update dominates the energy consumption of XYLS. Therefore, XYLS has poor performance with multiple actors. By contrast, the growth of actors has smaller impact on ERS, TS and TSR. It is because the search strategy is developed based on flooding, such that multiple actors can receive the search packet in one search attempt. Nevertheless, the growth of actors increases the average number of search attempts and hence increases the energy consumption. In addition, with the help of the redundancy reduction scheme, the energy consumption in TS-R performs better with more actors.

**Figure 15:**
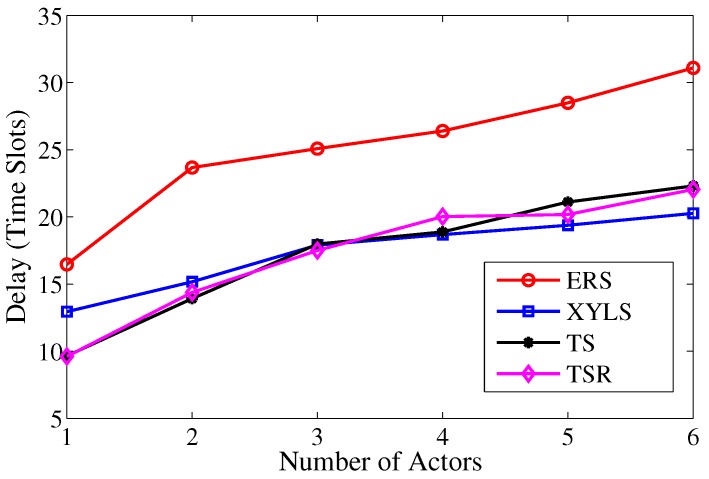
The search delay over the number of actors.

As shown in Figure 15, the search delay in TS is less than that in XYLS when there are fewer actors and becomes larger than that in XYLS when there are four or more actors. The reason for this is that the increase of actors can lead to additional search attempts, which generates more search delay than that in XYLS. The redundancy reduction scheme has no influence on the search phase, thus the search delay in TS and TSR are almost the same.

## 7. Conclusions

In this paper, we consider the problem of transmitting the event report to the mobile actor in wireless sensor and actor networks. By exploiting the trail information left by the mobile actor, we propose a trail-based search strategy (TS) that consists of search phase and chase phase. The search phase is successful when either the actor or its footprints are discovered, then the packets are forwarded along the trail until it is received by the actor in the chase phase. The redundancy reduction scheme based on TS (TS-R) is proposed to reduce the transmission redundancy by using local trail information. The theoretical analysis derives the condition to guarantee the trail connectivity, and proves that TS can reduce search delay and energy consumption by comparing with ERS. Simulation results verify the efficiency of the TS strategy.

TS is a reasonable solution to handle actor mobility in WSANs. It scales well in the scenarios with multiple actors. Moreover, It has better performance than ERS since it increases the success rate of search attempts by taking advantages of footprint information. Compared with location services, TS performs better in the event-driven applications where the data transmission is initiated only when the event occurs. It is an interesting issue to combine the trail-based mechanism with location service which will be studied in future works.

## Figures and Tables

**Table 1:** Variables List.

Symbol	Description
$N_{s}$	The number of sensor nodes in the network area
$\left(x_{i},y_{i}\right)$	The coordinate of node *i*
*H*	The side length of the network area
*L*	The side length of a grid
*R*	The transmission range of nodes
$T_{u}$	The update interval of the actor
ε	The threshold value of trail age
$\theta_{i}$	The footprint left at node *i*
$\alpha_{i}$	The age of footprint $\theta_{i}$
$d_{i,j}$	The distance between sensor node *i* and *j*
N(i)	The neighbors set of sensor node *i*
$N(A_{\theta })$	The neighbors set of the actor when it updates footprint θ
$\tau_{A}$	The waiting time of the actor
$\tau_{P}$	The waiting time of packet
*S*	The initial TTL value
